# A pilot study of measuring emotional response and perception of LLM-generated questionnaire and human-generated questionnaires

**DOI:** 10.1038/s41598-024-53255-1

**Published:** 2024-02-02

**Authors:** Zhao Zou, Omar Mubin, Fady Alnajjar, Luqman Ali

**Affiliations:** 1 School of Computer, Data and Mathematical Science, Western Sydney University, Sydney, Australia; 2 College of Information Technology, United Arab Emirates University, Al Ain, United Arab Emirates

**Keywords:** Psychology, Health care

## Abstract

The advent of ChatGPT has sparked a heated debate surrounding natural language processing technology and AI-powered chatbots, leading to extensive research and applications across various disciplines. This pilot study aims to investigate the impact of ChatGPT on users' experiences by administering two distinct questionnaires, one generated by humans and the other by ChatGPT, along with an Emotion Detecting Model. A total of 14 participants (7 female and 7 male) aged between 18 and 35 years were recruited, resulting in the collection of 8672 ChatGPT-associated data points and 8797 human-associated data points. Data analysis was conducted using Analysis of Variance (ANOVA). The results indicate that the utilization of ChatGPT enhances participants' happiness levels and reduces their sadness levels. While no significant gender influences were observed, variations were found about specific emotions. It is important to note that the limited sample size, narrow age range, and potential cultural impacts restrict the generalizability of the findings to a broader population. Future research directions should explore the impact of incorporating additional language models or chatbots on user emotions, particularly among specific age groups such as older individuals and teenagers. As one of the pioneering works evaluating the human perception of ChatGPT text and communication, it is noteworthy that ChatGPT received positive evaluations and demonstrated effectiveness in generating extensive questionnaires.

## Introduction

Recent years have witnessed the prevalence of the application of chatbots and natural language processing (NLP) technology in various fields. To be specific, NLP refers to the ability of the computer to understand and generate human-like texts ^1^. The latest deep learning tool ChatGPT released by OpenAI in 2021 ^2^ is now regarded as an artificial intelligence-powered (AI-powered) tool that could conduct human work ^3^. As a language model, Generative Pre-trained Transformer (GPT) is an example of NPL development ^4^. As a variant of GPT-3, ChatGPT is designed to generate human-like text naturally and to finalise general-purpose conversations ^5^. Human–computer interaction contributes to improving the user experience of the GPT model by designing interfaces that are both intuitive and user-friendly, facilitating easier interaction with GPT models and comprehension of their responses for users ^6^. At the same time, GPT offers researchers a novel approach to investigate users’ responses to computers and machines.

Due to its exceptional language-generating ability, ChatGPT has been extensively studied by researchers across various fields, including but not limited to education ^7^, healthcare ^8^, cybersecurity ^9^, and AI development ^10^. There is ongoing research into the impact of ChatGPT on users’ emotions, some studies suggest that emotional development in chatbots aims at providing more engaging and supportive interactions between users and machines. Meng and Dai compared emotional support from chatbot and human partners and stated that the AI chatbots increasingly engaged in emotional conversations with humans ^11^. Uludağ stated that although ChatGPT does not have emotions, it can recognize the users’ emotions and respond appropriately in human-like language ^12^. Besides, based on emotional contagion theory, Xu and other experts also believe that with the proper expectations and control levels over the emotions of the users, AI-powered chatbots could provide a positive influence on users’ emotions ^13^.

The primary motivation driving this research stems from the growing prevalence and significance of AI-powered chatbots, particularly represented by ChatGPT, in a wide array of applications. As the deployment of these AI-powered language models becomes increasingly pervasive, it is crucial to comprehensively understand their impact on human–computer interaction experiences ^14^. In this context, our study seeks to investigate the specific implications of integrating ChatGPT into an academic research questionnaire, with a particular emphasis on revealing users’ emotions and their interactions with AI-powered language model texts and communication.

Our investigation was motivated by existing literature, such as the work of Fischer, Kret, and Broekens, which suggests that females tend to react more sensitively than males when detecting emotions ^15^. Additionally, a study conducted in 2016 by Deng et al. ^16^ explored gender differences in emotional expression and revealed that women often report more intense feelings. Building upon these established findings, our study aims to explore the gender differences in response to AI-generated text and human-generated text. Therefore, in recognition of the importance of gender-based considerations in technology adoption and user experiences, our study also seeks to explore whether there exists gender-based differences in users' responses to the introduction of ChatGPT. Through a scientific analysis of users' interactions and emotion-detecting result, we hope to uncover any different patterns that might be obvious in how male and female users perceive and engage with the chatbot within the academic research questionnaire environment. Ultimately, we aim to advance our understanding of the complex interaction between artificial intelligence and human, thereby fostering the development of more user-friendly and empathetic AI-powered solutions.

In the designed study, ChatGPT is used to generate a copy of the questionnaire contents. ChatGPT was selected as a tool of generating content is because it is a language model trained on a massive amount of data, which allows ChatGPT to generate grammatically correct and human-like text ^5^. Additionally, ChatGPT can generate text on a wide range of topics, making it a multipurpose tool for tasks such as writing, summarizing, and responding to user inquiries ^17^. ChatGPT was utilized to generate the questionnaire for the present study, as it is capable of comprehending the conversation’s context and learning from previous interactions.

The participants are invited to complete two questionnaires with the same content, and the questionnaires are formulated by humans and ChatGPT separately. And their emotional changes are recorded by an Emotion Detecting Model. The study finds that ChatGPT plays a favourable role in improving participant’s happiness levels and reducing their sadness levels. What’s more, the study illustrates that female users tend to be more responsive to the introduction of ChatGPT. These results suggest that ChatGPT has significant potential for application in research domains and human–computer interaction contexts.

The subsequent sections of this paper will be structured as follows: section “Introduction” will reveal the methodology, including the generation of questionnaires and the development of the experimental setting, the design and implementation of the emotion-detecting model, the recruitment of the participants, and the methods to avoid ethics issues and reduce bias. Section “Methodology” will illustrate the data analysis, including the source data display, visualised graphs and results obtained from the data analysis. Section “Discussion” will provide a discussion of the findings, including an analysis of their implications and potential contributions to the existing literature, as well as an evaluation of the limitations of the study.

## Methodology

### Research design

The project is approved by Western Sydney University Human Research Ethics Committee (HREC Approval Number: H15278). All experiments were performed in accordance with relevant guidelines and regulations. Informed consent was obtained all from all subjects and/or their legal guardian(s) to participate in publishing the information and images in an online open-access publication.

The questionnaires are an effective method for investigating the emotional perception of ChatGPT as they provide a means to directly compare the emotional responses of participants during interactions with a human and with an AI-powered chatbot. By administering different questionnaires, one designed by a human and the other by human-ChatGPT, researchers can examine differences in emotional perception. Furthermore, while participants are working on the questionnaires online, their positions are relatively stable, therefore, their facial expressions can be captured by an internally installed emotion detection model. This allows for an objective measure of emotional response to be collected alongside the subjective measures provided by the questionnaires. The use of an emotion detection model provides researchers with additional insights into the emotional experiences of participants, allowing for a more comprehensive understanding of the emotional impact of ChatGPT.

In order to evaluate the influence of ChatGPT on users’ emotions, we designed two versions of the online questionnaire on Qualtrics. Questionnaire version 1 (V1) is formulated by “Humans” only, and questionnaire version 2 (V2) is formulated by “Humans and ChatGPT”, i.e., ChatGPT rephrased the survey questions created by humans and produced a human-ChatGPT version. The type of all questions is Single Choice, and the choices are completely the same (Table 1). However, the questions of the two questionnaires are created by human and ChatGPT separately. But the question contents share the same meaning. To be specific, after creating all questions in V1, we inserted requests in ChatGPT one by one:

Researcher: Please modify this question in an academic research questionnaire, the question is “……”.

ChatGPT provides a list of revised questions.

**Table 1:** Question contents of questionnaire V1 and V2.

Questionnaire V1 (Human-created)	Questionnaire V2 (ChatGPT-created)
What is your gender?	With respect, may I know your gender?
What is your age group?	To help us understand your experiences, can you tell us which age group you belong to?
How do you complete this questionnaire?	Can you let us know if you used any assistance in completing this questionnaire?
Do you have children?	May I know if you have any children?
Where do you live?	Can you tell me about your current living situation?
Do you remember what you ate for dinner yesterday?	Can you recall what you had for dinner yesterday?
Do you remember your date of birth?	Can you recall your date of birth?
Do you talk with your families more than 3 times a week?	Can you let me know if you talk with your family more than 3 times a week?
Do you talk with your neighbours more than 3 times a week?	Can you let me know if you talk with your neighbours more than 3 times a week?
Do you go out for walking more than 3 times a week?	Can you let me know if you go out for a walking more than 3 times a week?
Do you do other physical exercises except walking?	May I know if you do any other physical activities besides walking?

The selection criteria are the sequence. We select the first revised one on the list. But if the first question is too long and the content length is over 30 words, we then switched to the second one.

### Emotion detecting model

**Figure 1:**
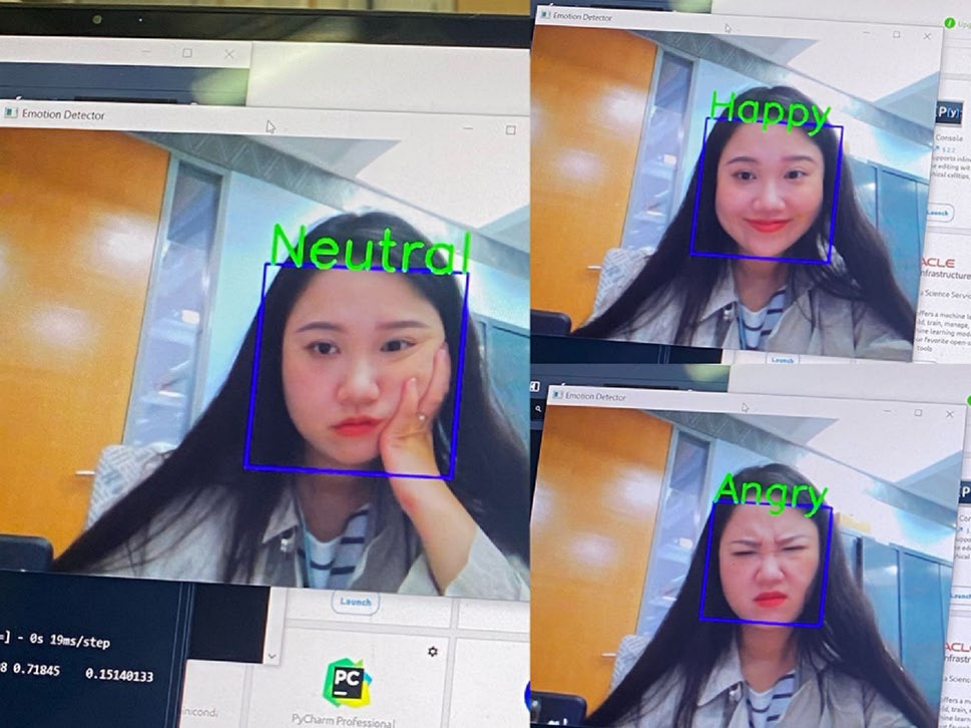
Sample display of emotion detecting model sample of source data.

The development of the adopted emotion-detecting model is implemented using the Python programming language. We have selected the YOLOv5 model as our chosen approach for emotion detection. YOLO (You Only Look Once) models are renowned for their real-time object detection capabilities, offering a balance between speed and accuracy ^18^. YOLOv5 utilized the PyTorch deep learning framework ^19,20^. The model was trained using an open dataset called the AffectNet dataset ^21^. AffectNet stands as an expansive dataset, encompassing approximately 400,000 images, neatly divided into eight distinct emotion categories: neutral, angry, sad, fearful, happy, surprise, disgust, and contempt. We conducted data augmentation specifically on a subset of the publicly accessible AffectNet dataset. The YOLOv5 model, a well-established and meticulously scrutinized framework in the domain, was employed for our endeavours. The model's prototype precisely curated 10,459 images for training purposes. The obtained results indeed exhibited promising. From these eight categories, five prevalent emotions, namely sad, angry, happy, neutral, and surprise, were selected for the training of the model employed in this study, as depicted in Fig. 1. The final model process entailed the use of 15,000 images. Detailed insights regarding the model's accuracy can be found in ^22^. However, to summarize, the mean Average Precision (mAP) at a threshold of 0.5 for the emotions of angry, happy, neutral, sad, and surprise is recorded as 0.905, 0.942, 0.743, 0.849, and 0.948, respectively. In the model, we designed 5 emotion classes: Angry, Happy, Neutral, Sad and Surprise. As indicated in Fig. 1, the model can detect and evaluate the major emotion of the current facial expression.

**Figure 2:**
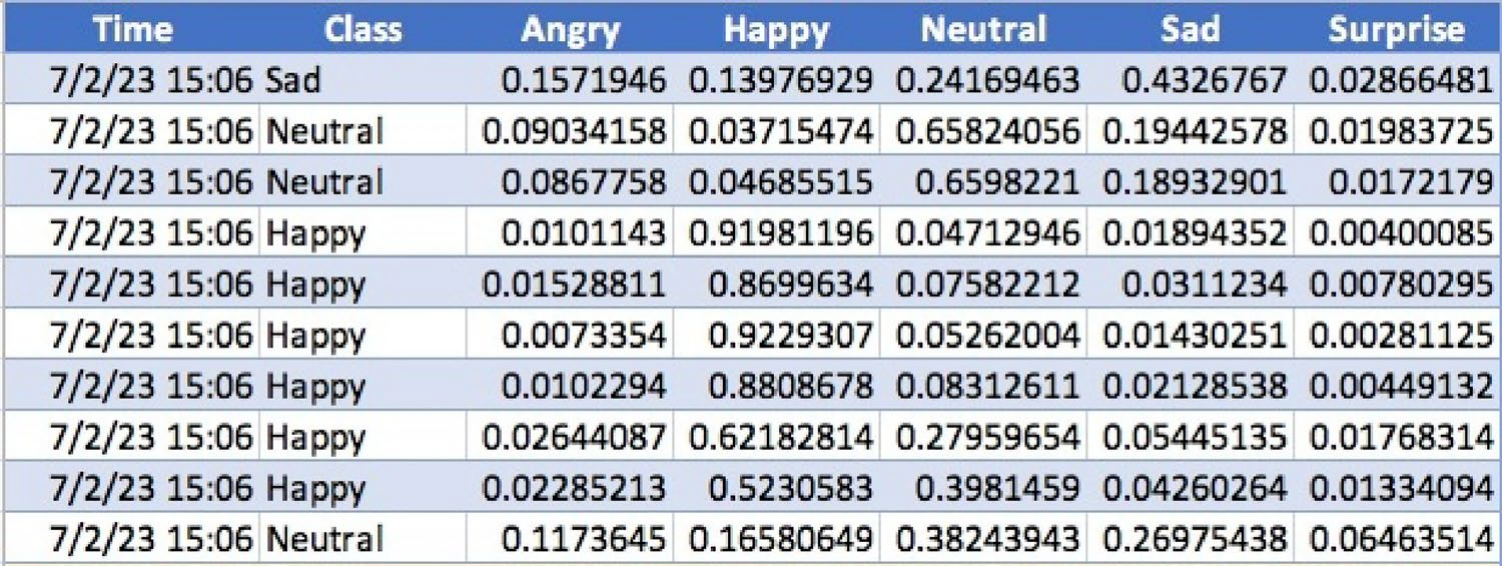
Sample of source data.

Every time the participant’s facial emotion change, a new record will be automatically created. Figure 2 is a piece of source data we get from the model. The model can calculate the percentage of each emotion and select the one that has the largest percentage. The time record of each emotion helps to match the questionnaire sections they are doing.

### Participants

A total 14 participants (7 female and 7 male) were randomly invited from the students in the United Arab Emirates University. The participants were required to finalise the online questionnaire in a classroom.

The participants will not be informed they are under emotion-detecting, which is to make sure they can act as naturally as possible. But when they finish the testing, the researcher will explain the test process and show them the emotion-detecting model. If the participants do not agree to use their data in the following experiments, we will remove all the related documents and data immediately. For those who give consent, their details are unidentifiable. Only the emotional data will be saved.

It is worth noting that according to a recent survey conducted by Statista ^23^, which indicated that the major age group of ChatGPT users falls within the 25–34 age range. Furthermore, a survey conducted by the Pew Research Center, as reported by Vogels ^24^, found that adults under 30 are more likely to be familiar with Chatbots and actively use ChatGPT in their daily lives. Considering these findings, we made a conscious decision to invite participants within this age group to participate in the pilot study. By focusing on the age group most representative of ChatGPT users, we aimed to ensure that the study's outcomes closely align with the user demographics and usage patterns.

### Bias reducing method

**Figure 3:**
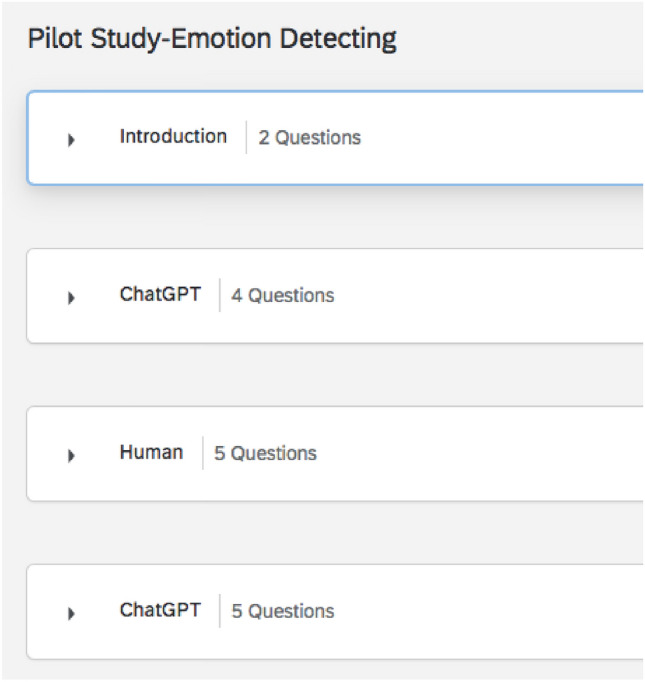
Display of questionnaire design.

To mitigate potential bias, we integrated the two versions of the questionnaire into a single instrument. To be specific, the testing questionnaire consists of different sections (as shown in Fig. 3): the ChatGPT section and the Human section. Each section is set a timer of 30 s. The participants cannot move to the next section until the time runs out. Therefore, the reading speed will not influence the testing progress speed. The blending of queries formulated by both ChatGPT and humans may result in participants exhibiting more authentic behaviour, as the queries become indistinguishable.

Meanwhile, a temporal tracking mechanism was integrated into the emotion detection model. It facilitates the association of emotional data with corresponding questionnaire segments.

When designing the questionnaire, we adopted neutral questions (Table 1). The decision to employ neutral and biography questions was driven by our objective to establish a standardized set of inquiries that could be compared objectively between the two distinct groups, devoid of any inherent biases or preconceived notions. Multimedia documents, encompassing text, images, sounds, or videos, tend to evoke diverse emotional responses in individuals exposed to them ^25,26^. Designing questions that evoke strong emotional responses would present challenges in distinguishing whether the observed emotional shifts were attributed to the question's nature, the generation method (via ChatGPT or human intervention), or other influencing factors. By integrating neutral questions, our aim was to mitigate potential biases that might be introduced by human surveyors. Additionally, within the “Extended research” section of our manuscript, we undertook supplementary efforts to investigate the utilization of both ChatGPT and human-generated questions across different age demographics. The incorporation of neutral questions allows for an assessment of ChatGPT's proficiency in generating questions across diverse contexts, maintaining a careful balance to avoid undue emotional influences.

### Data collection

The dataset comprises 8672 instances of emotion data associated with ChatGPT and 8797 instances associated with human responses, collected from a total of 14 participants. On average, each participant contributed data points within the range of approximately 1000 to 1800 points. Generally, the prevailing emotion observed in both V1 (ChatGPT version) and V2 (Human version) questionnaires is “Neutral” as shown in Table 2. The frequency of emotion in the female group (Table 3) and the male group (Table 4) has a slight difference.

**Table 2:** Average emotion frequency (general).

Class	Angry	Happy	Neutral	Sad	Surprise
ChatGPT	0.087	0.068	0.664	0.155	0.026
Human	0.095	0.047	0.654	0.172	0.032

**Table 3:** Average emotion frequency (female).

Class	Angry	Happy	Neutral	Sad	Surprise
ChatGPT	0.0777	0.0849	0.6801	0.1362	0.0212
Human	0.0886	0.0430	0.6928	0.1521	0.0235

**Table 4:** Average emotion frequency (male).

Class	Angry	Happy	Neutral	Sad	Surprise
ChatGPT	0.0987	0.0449	0.6430	0.1803	0.0331
Human	0.1030	0.0506	0.6101	0.1952	0.0411

**Figure 4:**
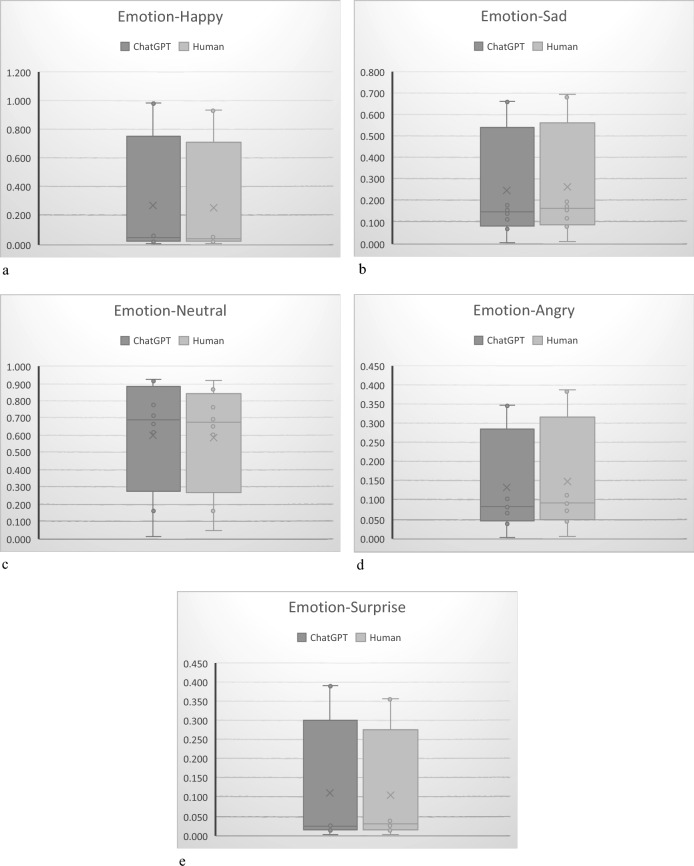
(**a**) Average value and distribution-happy. (**b**) Average value and distribution-sad. (**c**) Average value and distribution-neutral. (**d**) Average value and distribution-angry. (**e**) Average value and distribution-surprise.

Based on the graphical representation of the average values and standard deviations of each emotion (Fig. 4a–e), the small standard deviations suggest that the data is more tightly clustered around the mean.

## Discussion

The data from this pilot study suggest some potential relationships between participants’ emotions toward the Human-made questionnaire and the ChatGPT-made questionnaire.

**Figure 5:**
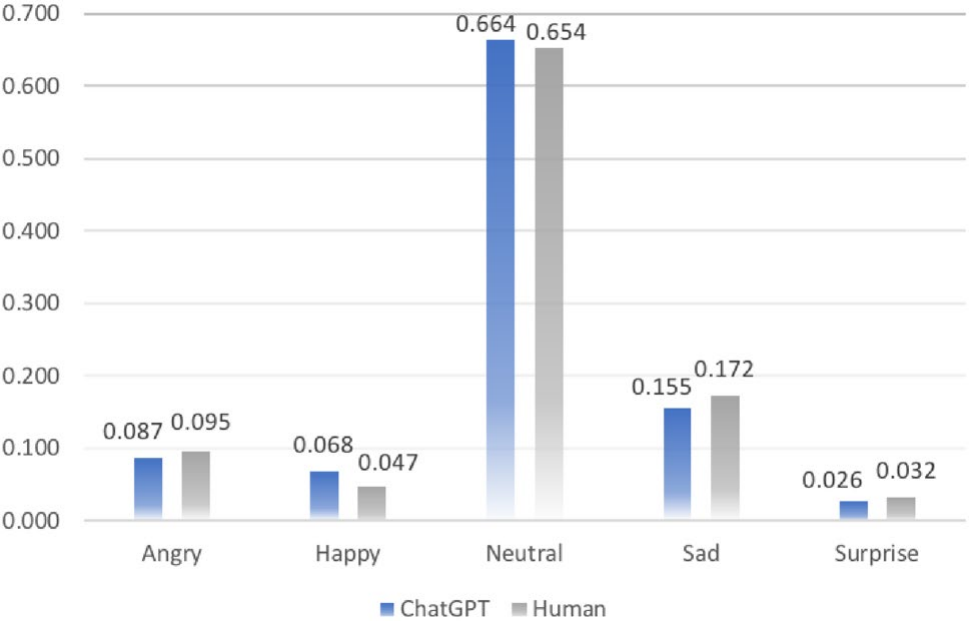
Average values of emotions.

While the dominant emotion illustrated from the data is “neutral” (with a mean value of approximately 0.6), some variations in the expression of other emotions were detected. As depicted in Fig. 5, the mean emotion values indicate that the participants experienced a higher level of happiness (ChatGPT Happy value: 0.068) when responding to the questionnaire formulated by ChatGPT compared with the questionnaire designed by humans (Human Happy value: 0.047). Meanwhile, they also experienced a lower level of sadness (ChatGPT Sad value: 0.155) when facing questionnaire V1, compared with questionnaire V2 (Human Sad value: 0.172).

**Figure 6:**
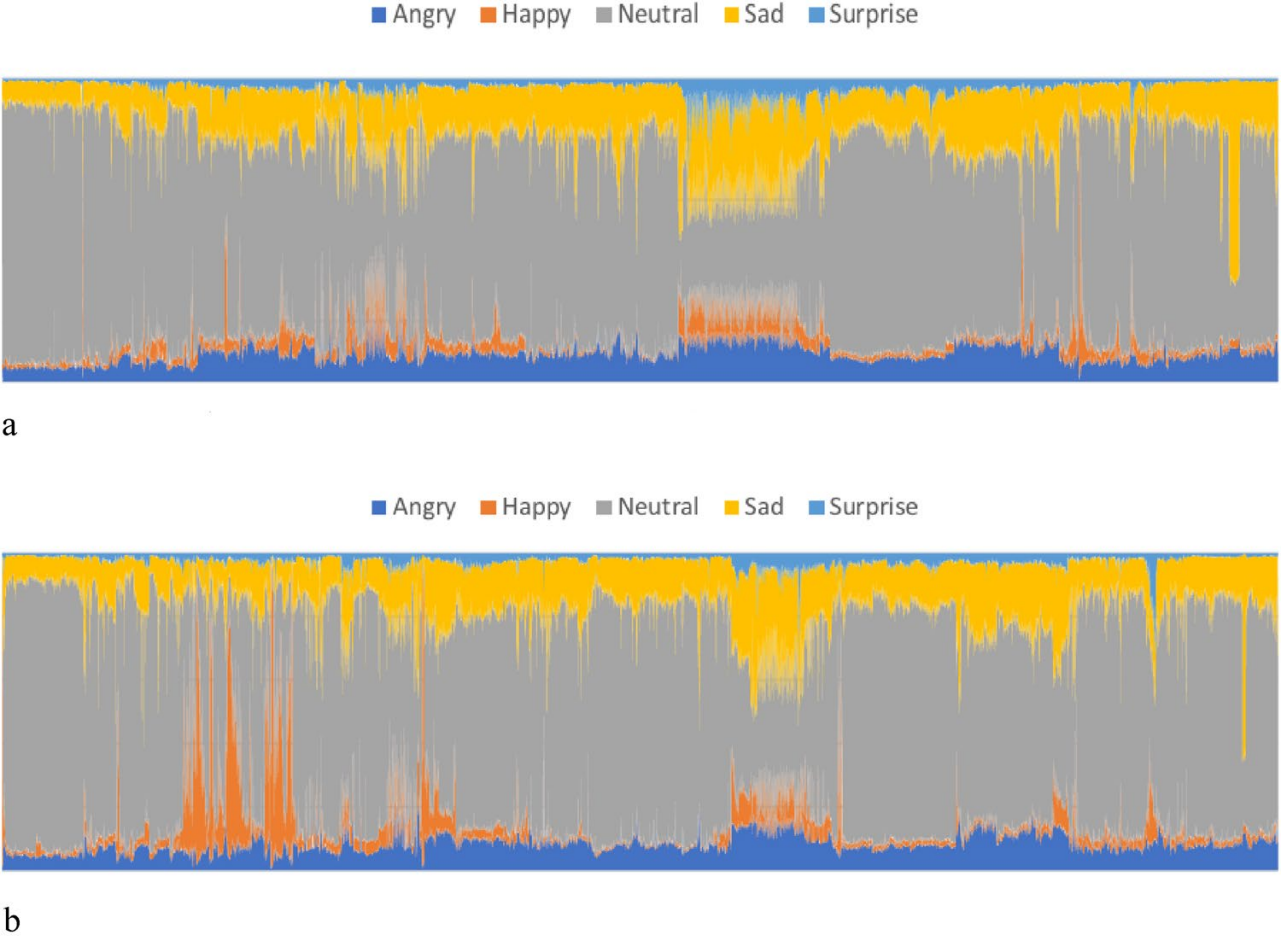
(**a**) Emotion changes during human-formulated questionnaire completion. (**b**) Emotion changes during ChatGPT-formulated questionnaire completion.

The emotional expressions of participants throughout the questionnaire completion process can be detected from the below graphs. A comparative analysis of the emotional dynamics captured in the graphs corresponding to the human-formulated questionnaire (Fig. 6a) and the ChatGPT-formulated questionnaire (Fig. 6b) indicates some differences. To be specific,

- The orange area which represents the emotion “Happy” in Fig. 6b is observed to be significantly larger than it is in Fig. 6a.
- The yellow area representing the emotion “Sad” is observed to become slightly smaller in Fig. 6b.
- And the grey area which stands for the emotion “Neutral” remains similar in size.

The mean values of the emotional expressions of participants of different genders can be detected from the below graphs. The graphical representation of the data indicates that significant gender differences are not observed. However, when examining specific emotions, such as Happiness, it is noteworthy that female participants exhibit greater sensitivity in their responses to different versions of the questionnaires.

**Figure 7:**
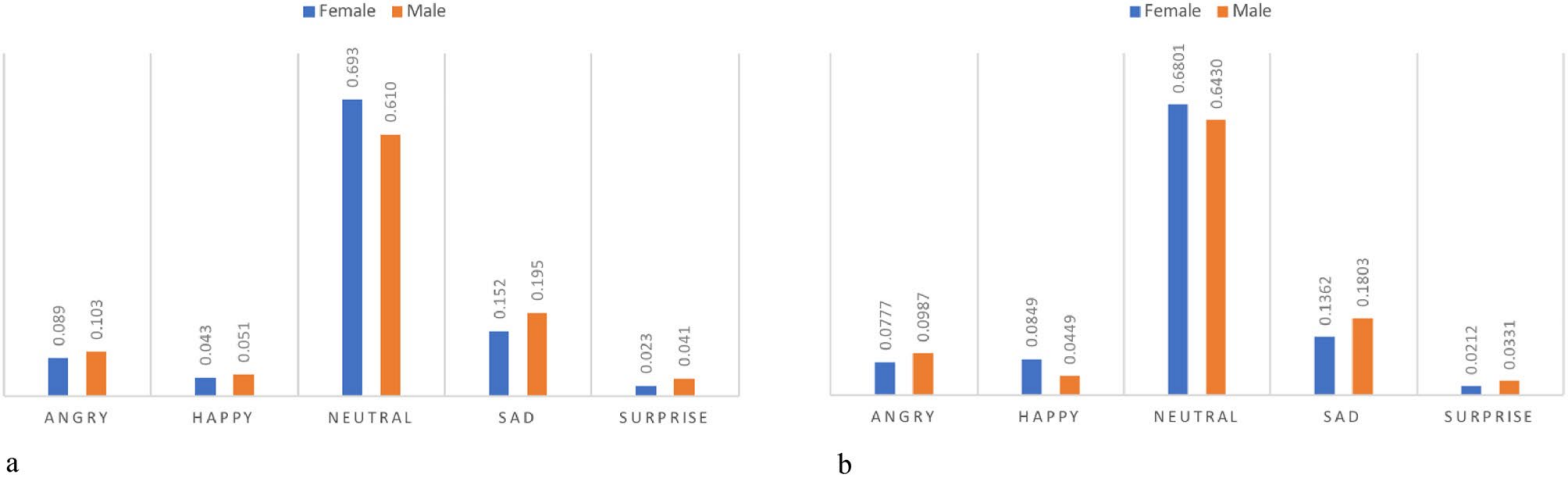
(**a**) Gender differences in emotion means on human questionnaire. (**b**) Gender differences in emotion means on ChatGPT questionnaire.

Specifically, female participants demonstrated higher levels of happiness than their male counterparts, with a ChatGPT Happy value of 0.085 and a Human Happy value of 0.043 (Fig. 7a, b). The observed difference in mean value between responding to the ChatGPT-formulated questionnaire and the Human-formulated questionnaire is estimated to be 0.042 (Mean value of ChatGPT minus the mean value of Human). In contrast, the difference in mean values for male participants was small, with a difference of only − 0.006 between the ChatGPT-formulated questionnaire and the Human-formulated questionnaire. In terms of the Angry emotion, the observed difference of female mean value between responding the ChatGPT-formulated questionnaire and the Human-formulated questionnaire is estimated to be − 0.011 (Mean value of ChatGPT minus the mean value of Human), while for males the difference number is − 0.004.

We performed a repeated measures Analysis of Variance (ANOVA) to compare measurements within participants for two types of questionnaires: Human-generated Questionnaire and ChatGPT-generated Questionnaire. We analysed the average emotion readings per participant across both questionnaire types, considering gender as a covariate. The results, presented in Table 5, revealed significant findings for some emotions such as surprise, anger, and sadness. Interestingly, the ANOVA results indicated no significant difference in user reactions between the Human-generated questionnaire and the ChatGPT-generated questionnaire.

**Table 5:** Summary of ANOVA analysis.

Emotion	Within subject effect: questionnaire type	Between subject effect: gender
Happy	F(1,12) = 0.76, p = 0.401	F(1,12) = 0, p = 0.983
Neutral	F(1,12) = 0.001, p = 0.992	F(1,12) = 2.55, p = 0.136
Sad	F(1,12) = 0.001, p = 0.981	F(1,12) = 3.68, p = 0.079
Angry	F(1,12) = 3.29, p = 0.095	F(1,12) = 3.72, p = 0.078
Surprise	F(1,12) = 0.75, p = 0.403	F(1,12) = 3.94, p = 0.071

**Figure 8:**
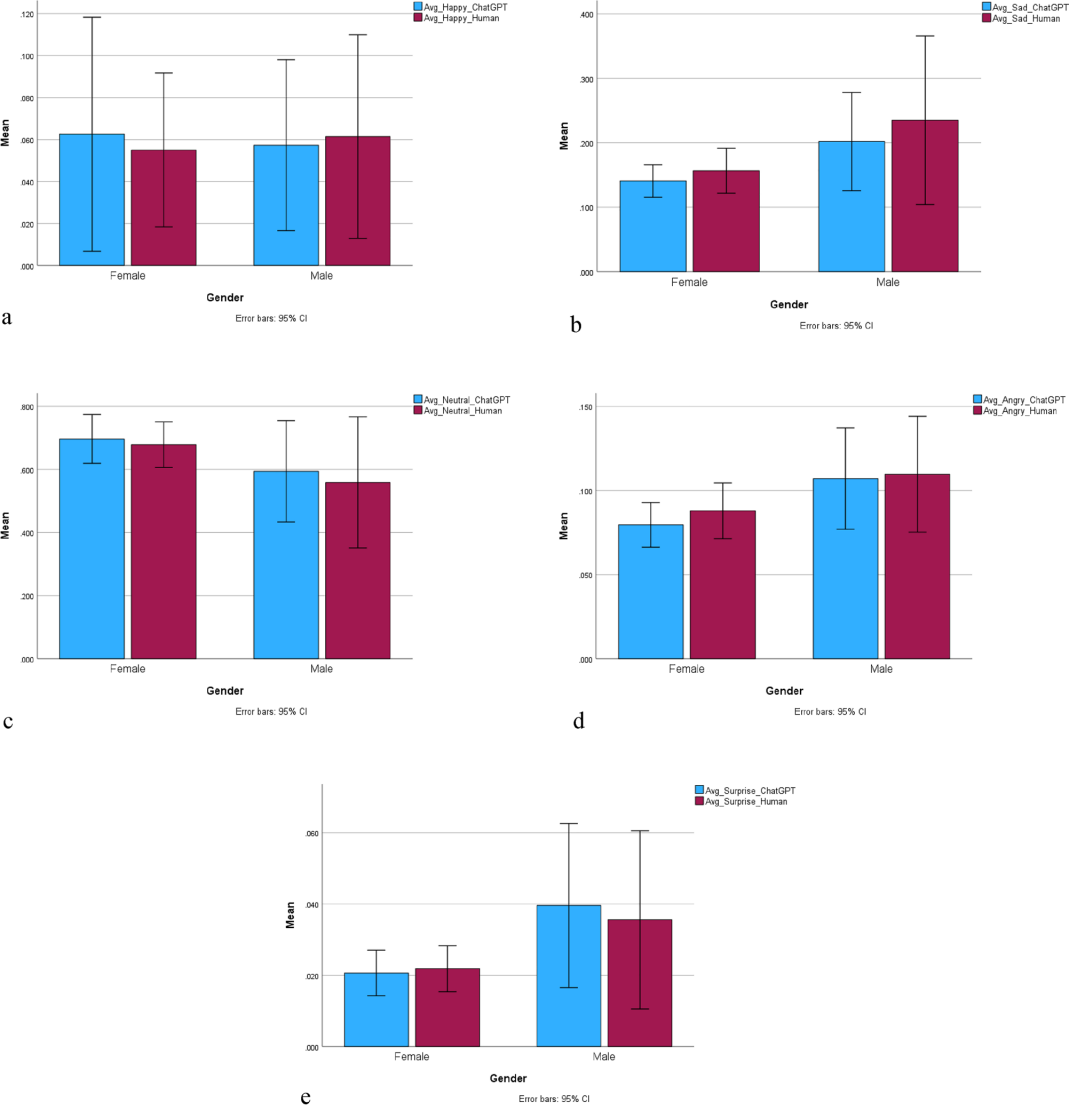
(**a**) Gender distribution-happy. (**b**) Gender distribution-sad. (**c**) Gender distribution-neutral. (**d**) Gender distribution-angry. (**e**) Gender distribution-surprise.

Based on a systematic literature review by Forni-Santos and Osório, it has been observed that women generally outperform men in recognizing a broad set of basic facial expressions ^27^. However, our ANOVA results, depicted in bar charts (Fig. 8a–e), indicate no significant gender effect, as evidenced by p-values exceeding 0.05. Forni-Santos and Osório specifically noted that there appears to be no huge gender-related differences in the recognition of happiness, while findings are more varied for other emotions, particularly sadness, anger, and disgust ^27^. Interestingly, our study reveals that male participants tend to exhibit greater expressiveness, particularly in relation to specific emotions such as sadness, anger, and surprise.

Upon careful analysis of the data, it is evident that the emotional perception of human and ChatGPT-generated questionnaires does not exhibit a significant difference. However, we have observed certain subtle trends that appear to favour ChatGPT's performance. Additionally, we have taken note of the fact that the ChatGPT questionnaire received positive evaluations and was not negatively perceived in comparison to the human-generated questionnaire. These findings provide valuable insights that support the potential applications of generative AI in content generation and questionnaire design.

Xu et al. ^13^ found AI chatbots positively influence emotions in business and retail, using a video-based experiment and subjective user questionnaires. In contrast, our study focuses on academia, using ChatGPT and a more objective emotional detection model to evaluate participants’ emotions and feedback while completing various agent-generated questionnaires.

### Extended research

To obtain a more comprehensive knowledge of the impact of AI-powered language models on individuals’ emotional and behavioural responses while completing questionnaires, we utilized the third version of the questionnaire (V3). To be specific, the study utilized three distinct versions of a questionnaire: Version 1 (V1) was a purely human-created version in which all the questions were generated solely by human researchers. Version 2 (V2) was a hybrid human-AI version, in which the ChatGPT model was employed to revise or rephrase the questions from V1, resulting in a modified version of the questionnaire. Finally, Version 3 (V3) was a pure AI-generated one, in which the researchers provided ChatGPT with specific criteria, including the desired functions and target participants, as well as necessary information, and allowed the model to automatically generate the questionnaire.

**Figure 9:**
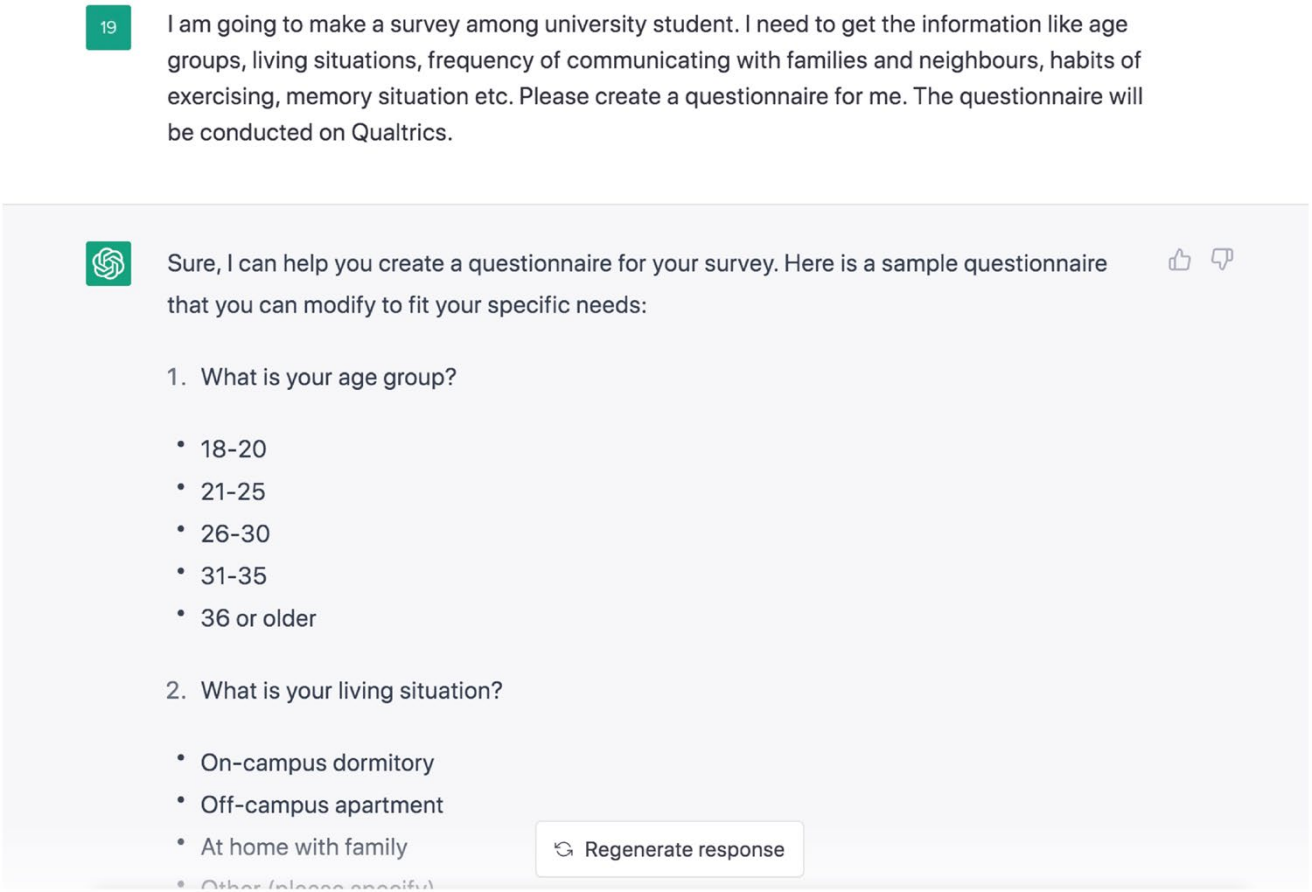
ChatGPT questionnaire results.

Demonstrated in Fig. 9 are the comprehensive specifications and corresponding outputs resulting from the request to generate a questionnaire using ChatGPT (the full version of the questionnaire provided in the appendix). The image demonstrates that ChatGPT is equipped to produce complex questionnaires, including multiple-choice options. The generated questionnaire was subsequently transferred to Qualtrics to create an online version for participants to complete.

The experiment was conducted using a methodology in which questions from all three questionnaire versions were combined into a single questionnaire sheet, with participants remaining unaware of the specific source of the questions they were responding to.

The findings of the extended experiment, as presented in Table 6, provide several noteworthy observations. Specifically, in terms of the emotions of happiness, and sadness, the average values obtained for Version 3 exhibit slight differences from those obtained for Versions 1 and 2. Nevertheless, it should be highlighted that the emotion of neutrality exhibits an approximate 0.4 decrease in Version 3 relative to its values in the other two versions. Moreover, the amount of this decrease appears to be shared by the minor emotions “Angry” and “Surprise”, which were also detected to a lesser extent in Versions 1 and 2. Both of the average values of “Angry” and “Surprise” in Version 3 are higher than those in Version 1 and 2.

**Table 6:** Average values of emotions responding three versions of questionnaire.

Emotions	Pure-human version (V1)	Human-AI version (V2)	Pure AI version (V3)
Angry	0.0954	0.0867	0.1223
Happy	0.0465	0.0678	0.0598
Neutral	0.6540	0.6642	0.6209
Sad	0.1724	0.1551	0.1442
Surprise	0.0318	0.0263	0.0538

The results observed may potentially result in the fact that in the absence of intervention by human researchers, some survey questions generated purely by the AI language model could be regarded as offensive to some extent. As presented in Table 7, notable differences between Versions 3 and Versions 1 and 2 were observed in several questions, particularly those concerning participants’ private experiences, for instance, memory levels. Versions 1 and 2, guided by human intervention, avoided directly asking about participants’ memory status, preferring to conclude their memory levels through questions about their ability to recall past events. However, Version 3 generated questions that more directly probed into participants' private details. Such questions may have contributed to the higher emotion values obtained for “Angry” and “Surprise” in Version 3.

**Table 7:** Comparisons of questions in V1/V2 and V3.

About memory level	V1 and V2	Do you remember your date of birth? Do you remember what you ate for yesterday’s dinner?
	V3	How would you describe your memory situation? Have you ever sought help for your memory?
About seeking help	V1 and V2	NA
	V3	If you sought help for memory, how did you seek help?
About counselling services	V1 and V2	NA
	V3	Have you ever received any counselling services? What type of counselling did you receive?

### Ethical statement

This pilot study has received ethical approval from the Western Sydney University Human Research Ethics Committee (Approval Number: H15278), acknowledging its alignment with our broader research on AI and humanoid agents’ impact on psychological well-being. All procedures complied with the committee’s guidelines and regulations. Rigorous informed consent was obtained from all participants and/or their legal guardians for both participation and the publication of information and images in an online open-access format, ensuring adherence to ethical standards of participant autonomy, privacy, and well-being.

## Discussion

The findings of this pilot study suggest that adopting an AI-powered Chatbot like ChatGPT may be a promising strategy for promoting the users’ satisfaction and happiness when it is manipulated by humans. The results of the study indicate that the involvement of ChatGPT in the questionnaire design is associated with an increase in users’ reported happiness levels, at the same time, the sadness level is also seen a decrease as evidenced by the data. Another notable finding of this study is that in terms of emotion Happiness, the female participants appeared to be more responsive to the introduction of ChatGPT, as indicated by a greater increase in their reported levels of happiness compared to male participants.

Notably, ChatGPT generated the questionnaire sorely without human intervention and did not draw the anticipated positive responses from the participants. Surprisingly, there was a slight increase in the expression of “Angry” and “Surprise” emotions. This outcome may have been rooted in certain questions in the AI-generated questionnaire, which could have been perceived as potentially offensive.

It is significant to highlight that the implementation of a research questionnaire that is created by humans with the assistance of AI-powered language models represents a superior approach for enhancing user satisfaction and fostering a sense of happiness. Furthermore, it was discovered that ChatGPT can offer a diverse range of responses when requests are proposed with more details. As illustrated in Table 8, it provides an example of the various responses produced by ChatGPT in different scenarios. The main task of ChatGPT is to generate appropriate responses to inquiries concerning the age groups of the participants. To complete this, three different scenarios have been established, including “young kids,” “university students,” and “older people.” While some of the responses provided by ChatGPT are universal and thus overlap across scenarios, specific recommendations personalised to each age group are also provided. Regarding the “young kids” group, the responses provided by ChatGPT are intentionally designed to be straightforward and easy to understand, as children may encounter difficulties in understanding complicated information. In the case of “university students,” the responses are formulated to be precise and clear, ensuring that adults can grasp the concept directly. When communicating with the “older people” group, ChatGPT is capable of generating responses that show a higher level of respect and sensitivity, thereby enhancing the overall user experience.

**Table 8:** ChatGPT provide responses based on different requests.

Ask ages from	ChatGPT responses
Young Kids	How old are you?
	When is your birthday?
	Are you 3, 4, or 5 years old?
	Can you show me with your fingers how old you are?
University Students	What is your age?
	How old are you?
	What year were you born?
	What is your date of birth?
Older People	What is your age?
	How old are you?
	What year were you born?
	May I ask what age group you belong to?

### Limitations

Although this study has generated significant findings and made notable contributions, it is crucial to acknowledge and address certain limitations for future research endeavours. First and foremost, the sample size employed in this study was relatively small, consisting of only 14 participants. We acknowledge the significance of adopting a larger sample size in order to obtain more reliable results and conclusions. However, we hope that as a pilot study, our present research findings, derived from a sample size of 14 participants, may make an elementary contribution to the field of measuring users’ emotional responses to ChatGPT. Also, it is worth mentioning that there is a number of 8 k–10 k data points were generated per participant. The primary objective of this paper is to conduct an introductory investigation into this topic. Future work aims to resolve this, and this has been indicated.

Moreover, the age range of the participants was restricted to individuals between 18 and 35 years old, potentially limiting the generalizability of the findings to a broader age demographic. Additionally, it is essential to recognize that the experiment was conducted exclusively in the United Arab Emirates, and as such, the results may be subject to cultural influences that are specific to this region. To enhance the robustness and applicability of future studies, it is recommended to incorporate larger and more diverse participant samples across multiple cultural contexts.

A potential research direction for future work may involve exploring the potential effects on user emotions by incorporating supplementary language models or chatbots, with a particular focus on other specific age demographics such as teenagers and older individuals.

## Conclusion

In conclusion, this pilot study aimed to investigate the impact of integrating an AI-powered chatbot, specifically ChatGPT, into an academic research questionnaire with a focus on users’ emotions. As one of the first works to evaluate human perception of ChatGPT text and communication, our study contributes to the literature development on human perception of AI-powered language model texts and communication. The findings revealed that users reported higher levels of happiness when utilizing the hybrid-formulated questionnaire, which combines chatbot-formulated questions with human intervention, compared to both the solely human-formulated and pure-AI-formulated questionnaires. Gender differences were not prominent, although male users demonstrated slightly higher responsiveness and expressiveness towards the chatbot compared to female users. Furthermore, ChatGPT displayed the ability to adapt responses according to different scenarios to enhance the user experience. Nevertheless, it is important to note that the study's restricted sample size, limited age range and cultural impacts impose limitations on the generalizability of the results. Overall, this pilot study provides a foundation for future research exploring the effectiveness of ChatGPT and other chatbots in improving user experiences. It is encouraging to observe that ChatGPT was not negatively evaluated and can be effectively utilized in generating lengthy questionnaires.

### Supplementary Information

Supplementary Information.

## Data Availability

The data that support the findings of this study are available from the corresponding author, Fady Alnajjar, upon reasonable request. The availability of the data is subject to certain conditions and restrictions to ensure data privacy, confidentiality, and adherence to ethical guidelines. To access the data, interested individuals or organizations may contact the corresponding author via email or postal mail. The corresponding author will review each request and provide the necessary information and data files, where feasible, to facilitate further analysis and replication of the study's findings. In the interest of promoting transparency and reproducibility, the corresponding author is committed to making the data available to qualified researchers and scientists.
