# Supplementary material for: A pilot study of measuring emotional response and perception of LLM-generated questionnaire and human-generated questionnaires
Source: Sci Rep. 2024 Feb 2;14:2781. doi: 10.1038/s41598-024-53255-1 (PMC10837442; doi:10.1038/s41598-024-53255-1)
Supplement: Supplementary file 1 — Supplementary Information. [file 41598_2024_53255_MOESM1_ESM.docx]

# Appendix I

**Full content of Pure-AI-Formulated Questionnaire**

| Researcher’s request:  I am going to make a survey among university student. I need to get the information like age groups, living situations, frequency of communicating with families and neighbours, habits of exercising, memory situation etc. Please create a questionnaire for me. The questionnaire will be conducted on Qualtrics. | |
| --- | --- |
| 1. What is your age group? | - 18-20 - 21-25 - 26-30 - 31-35 - 36 or older |
| 1. What is your living situation? | - On-campus dormitory - Off-campus apartment - At home with family - Other (please specify) |
| 1. How often do you communicate with your family? | - Every day - A few times a week - Once a week - A few times a month - Rarely |
| 1. How often do you communicate with your neighbours? | - Every day - A few times a week - Once a week - A few times a month - Rarely |
| 1. How often do you exercise? | - Every day - A few times a week - Once a week - A few times a month - Rarely |
| 1. How would you describe your memory situation? | - Excellent - Good - Average - Poor - Very poor |
| 1. Have you ever sought help for your memory? | - Yes - No |
| 1. If you answered yes to the previous question, how did you seek help? | - Self-help resources - Doctor/therapist - Family/friends - Other (please specify) |
| 1. Have you ever received any counselling services? | - Yes - No |
| 1. If you answered yes to the previous question, what type of counselling did you receive? | - Academic counselling - Career counselling - Mental health counselling - Other (please specify) |

**Appendix II**

**Full content of ChatGPT changes responses in various scenarios**

| **Young Kids** | 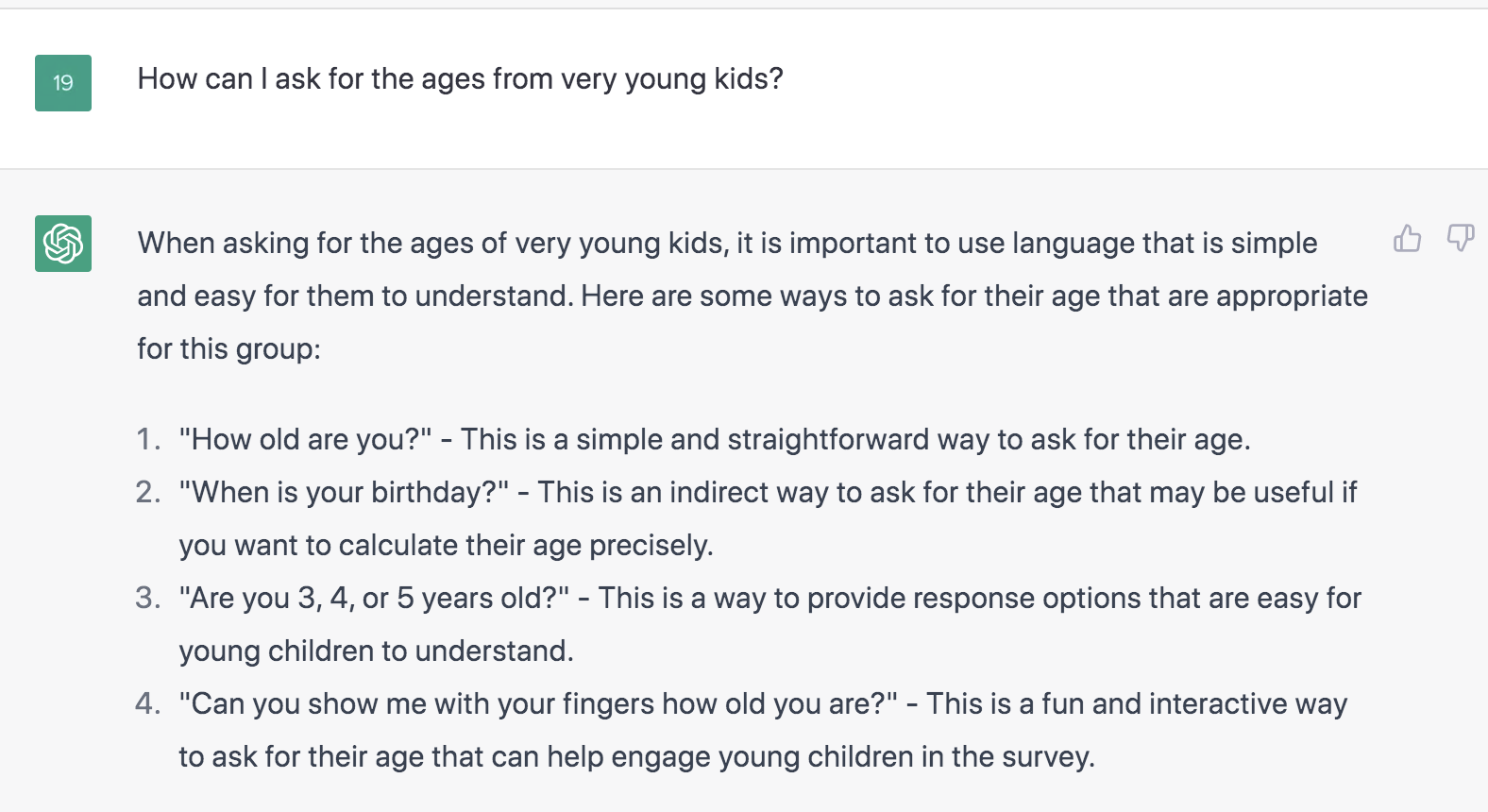 |
| --- | --- |
| **University Students** | 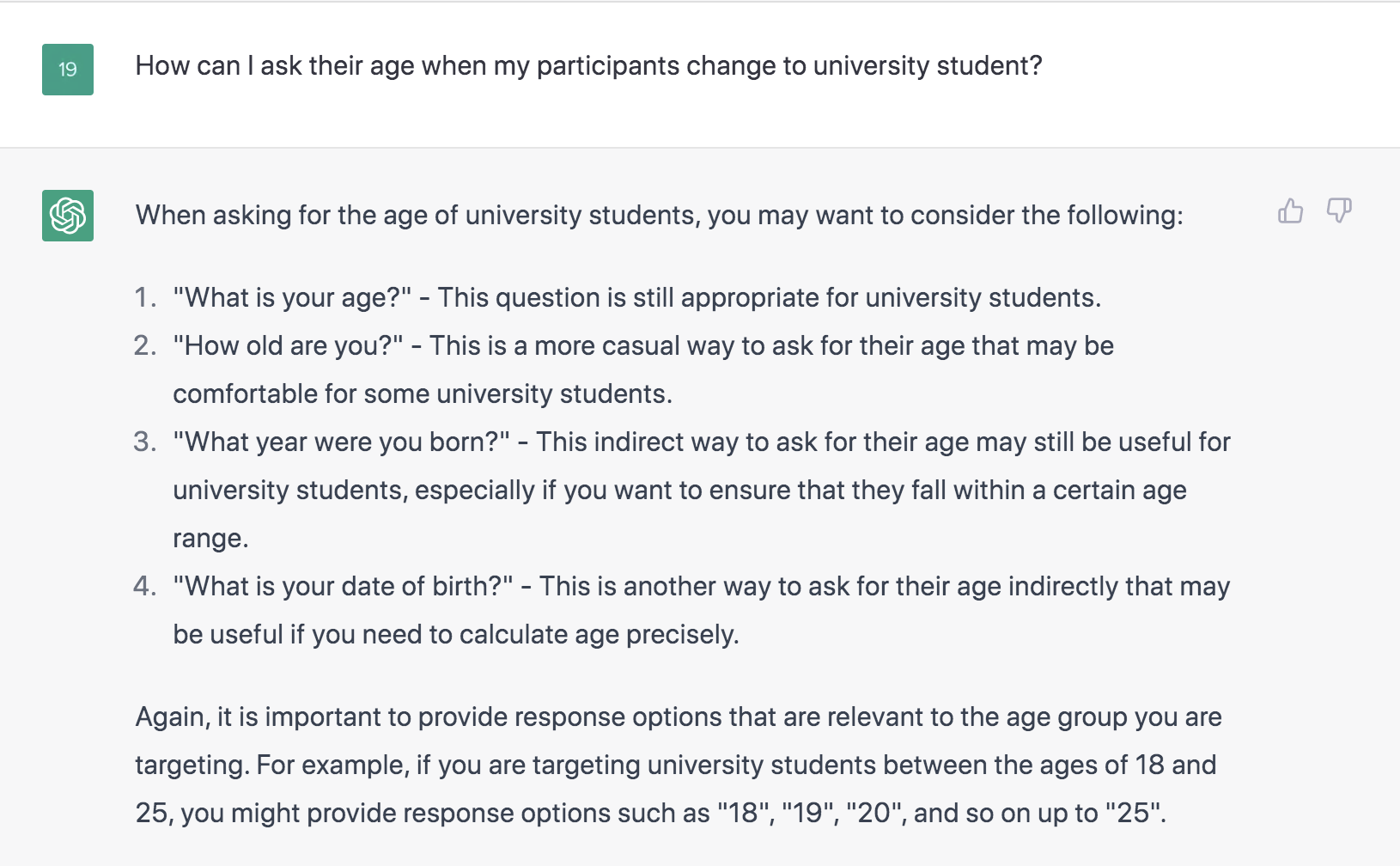 |
| **Older People** | 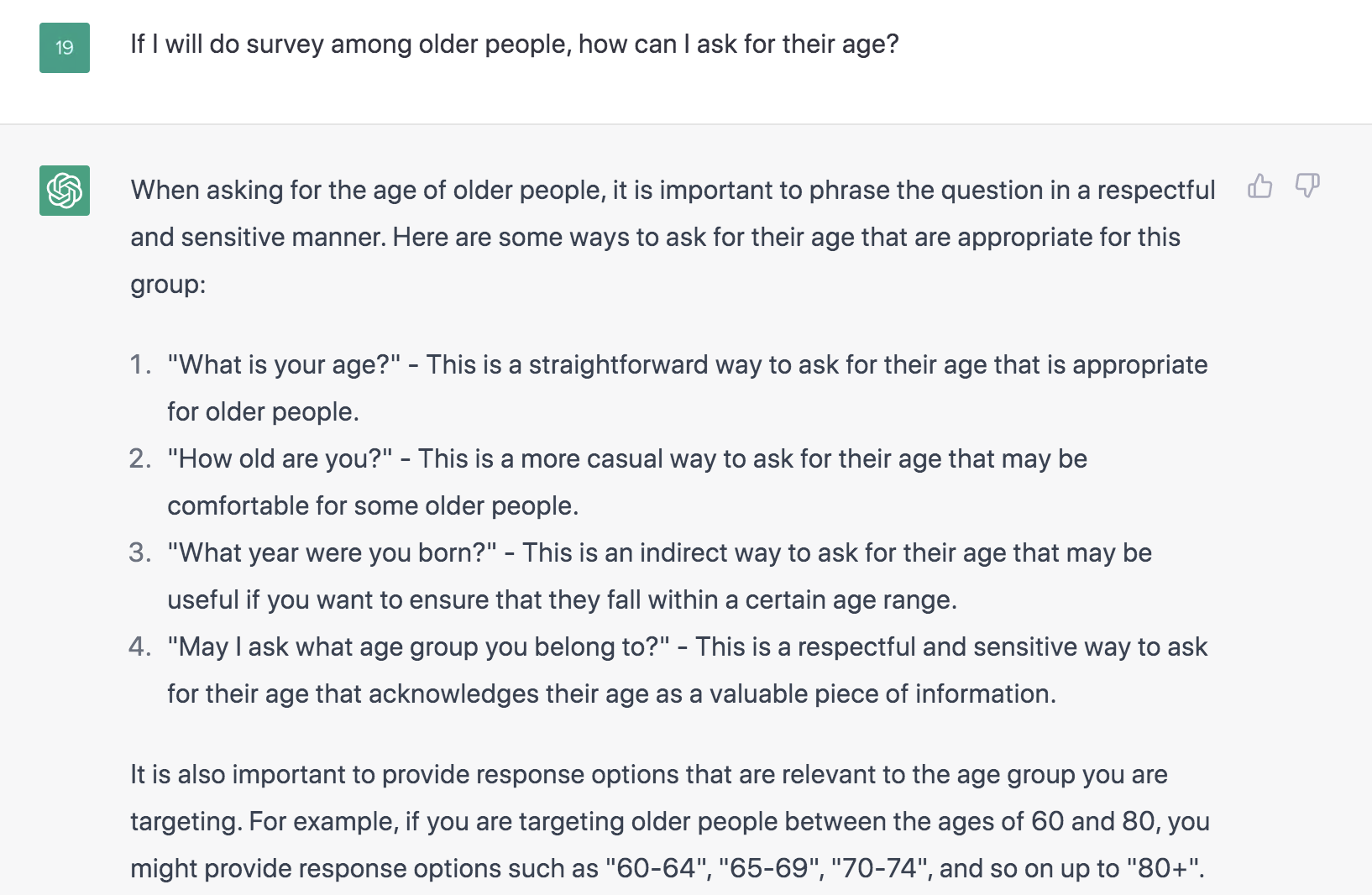 |
